# A Developmental and Molecular View of Formation of Auxin-Induced Nodule-Like Structures in Land Plants

**DOI:** 10.3389/fpls.2016.01692

**Published:** 2016-11-11

**Authors:** Ryan Hiltenbrand, Jacklyn Thomas, Hannah McCarthy, Karl J. Dykema, Ashley Spurr, Hamilton Newhart, Mary E. Winn, Arijit Mukherjee

**Affiliations:** ^1^Department of Biology, University of Central Arkansas, ConwayAR, USA; ^2^Bioinformatics and Biostatistics Core, Van Andel Research Institute, Grand RapidsMI, USA

**Keywords:** nodule-like structures, auxin, rice, *Medicago truncatula*, common symbiotic pathway, RNA sequencing, *Azorhizobium caulinodans*

## Abstract

Several studies have shown that plant hormones play important roles during legume–rhizobia symbiosis. For instance, auxins induce the formation of nodule-like structures (NLSs) on legume roots in the absence of rhizobia. Furthermore, these NLS can be colonized by nitrogen-fixing bacteria, which favor nitrogen fixation compared to regular roots and subsequently increase plant yield. Interestingly, auxin also induces similar NLS in cereal roots. While several genetic studies have identified plant genes controlling NLS formation in legumes, no studies have investigated the genes involved in NLS formation in cereals. In this study, first we established an efficient experimental system to induce NLS in rice roots, using auxin, *2,4-D*, consistently at a high frequency (>90%). We were able to induce NLS at a high frequency in *Medicago truncatula* under similar conditions. NLS were characterized by a broad base, a diffuse meristem, and increased cell differentiation in the vasculature. Interestingly, NLS formation appeared very similar in both rice and *Medicago*, suggesting a similar developmental program. We show that NLS formation in both rice and *Medicago* occurs downstream of the common symbiotic pathway. Furthermore, NLS formation occurs downstream of cytokinin-induced step(s). We performed a comprehensive RNA sequencing experiment to identify genes differentially expressed during NLS formation in rice and identified several promising genes for control of NLS based on their biological and molecular functions. We validated the expression patterns of several genes using reverse transcription polymerase chain reaction and show varied expression patterns of these genes during different stages of NLS formation. Finally, we show that NLS induced on rice roots under these conditions can be colonized by nitrogen-fixing bacteria, *Azorhizobium caulinodans*.

## Introduction

Root nodule formation is the result of two tightly coordinated processes: an organogenic process leading to nodule formation and an infection process mediating bacterial colonization. A complex signal exchange between the host plant and bacteria are required to initiate this symbiosis. The host roots secrete flavonoids, which are recognized by rhizobia. In response, rhizobia secrete lipochitooligosaccharides called Nod factors (NFs), triggering the nodule developmental process (Oldroyd and Downie, 2008; Venkateshwaran et al., 2013). Interestingly, a similar signal exchange is also required for the initiation of arbuscular mycorrhizal (AM) symbiosis (Maillet et al., 2011). Recognition of NFs by LysM receptor kinases at the plasma membrane triggers the signaling pathway leading to bacterial infection and nodule organogenesis. This signal transduction pathway includes a leucine-rich repeat receptor-like kinase (SYMRK/DMI2), cation channels (POLLUX and CASTOR), nucleoporins (NUPs and NENA), a calcium calmodulin-dependent protein kinase (CCaMK/DMI3), and a nuclear-localized coil-coil protein (CYCLOPS/IPD3). Further downstream, the GRAS transcription factors (TFs), NSP1 and NSP2, and the transcriptional regulator NIN are required for initiation of nodule organogenesis (Oldroyd and Downie, 2008; Venkateshwaran et al., 2013). Mutants in *CCaMK* (*DMI3*), *NSP1, NSP2*, and other genes are affected in most responses to NFs and are unable to form nodules (Oldroyd and Downie, 2008; Venkateshwaran et al., 2013). Interestingly, *DMI3, IPD3* and other genes are also required for AM symbiosis, leading to the concept of a shared “common symbiotic pathway (CSP)” between these two symbioses (Oldroyd and Downie, 2008; Venkateshwaran et al., 2013). Furthermore, some of the genes from this pathway are also required for AM symbiosis in rice (Chen et al., 2007, 2008, 2009; Gutjahr et al., 2008). This makes the prospect of engineering nitrogen fixation in cereals promising (Charpentier and Oldroyd, 2010; Rogers and Oldroyd, 2014; Mus et al., 2016).

Several studies have shown that plant hormones play key roles throughout nodule organogenesis in the integration of developmental and environmental signaling cues during nodule development (Ding and Oldroyd, 2009; Mukherjee and Ané, 2011b; Ryu et al., 2012; Bensmihen, 2015). For instance, the interplay of auxin and cytokinin plays a major role in nodule organogenesis (Suzaki et al., 2013). Cytokinins positively regulate nodulation-related expression of *NSP2* and *NIN* genes, and the expression of *PINs* that lead to local auxin accumulation in the primordial dividing cells leading to initiation of nodule organogenesis (Mathesius et al., 2000; Plet et al., 2011; Suzaki et al., 2012; Ng et al., 2015). The cytokinin receptors *LHK1/CRE1* are involved in nodulation, as loss-of-function mutations of these genes are impaired in nodule formation. However, gain-of-function mutants in these genes lead to formation of spontaneous nodules or nodule-like structures (NLS) in absence of bacteria (Murray et al., 2007; Tirichine et al., 2007). These findings uncouple bacterial infection from organogenesis, demonstrating that cortical cell divisions can be activated in plant roots leading to formation of NLS whether or not bacterial invasion occurs (Desbrosses and Stougaard, 2011). Similar to cytokinins, auxins also trigger cortical cell divisions leading to the formation of NLS even in the absence of bacteria. For instance, application of auxin transport inhibitors (ATI) such as naphthylphthlamic acid (NPA) and 2,3,5-triiodobenzoic acid (TIBA) induces NLS formation in roots of *Medicago sativa, Medicago truncatula*, and sweet clover (Hirsch et al., 1989; Wu et al., 1996; Rightmyer and Long, 2011). Structurally, these are comparable to the empty nodules stimulated by non-invasive bacteria such as the *exoA*^-^ mutant of *Sinorhizobium meliloti* at the histological and molecular level (Hirsch et al., 1989). However, NLS can be distinguished from rhizobia-induced nodules by their diffuse meristem and vascular tissue differentiation at the proximal and central part of the structure (Wu et al., 1996; Rightmyer and Long, 2011). NLS can also be distinguished from lateral roots based on their origin and anatomy. Lateral roots have a clearly defined apical meristem and originate from pericycle cells (Desbrosses and Stougaard, 2011). Nevertheless, all these processes result from meristem activity initiated by dedifferentiation of cells next to the vascular system. It is likely there may be a significant overlap in the genetic pathways controlling the formation of these root structures. Surprisingly, genetic analysis of pathways controlling initiation of nodule development and lateral root formation has not identified an obvious overlap (Desbrosses and Stougaard, 2011). Also, very few legume mutants have been identified that directly affect hormonal pathways (Desbrosses and Stougaard, 2011; Mukherjee and Ané, 2011b), possibly due to genetic redundancy. One option to elucidate the genetic mechanism in hormone-dependent nodule organogenesis is to study NLS formation. Interestingly, in many cereals such as rice, wheat, and maize, addition of phytohormones also stimulate NLS formation (Ridge et al., 1993; Kennedy et al., 1997; Christiansen-Weniger, 1998; Narula et al., 2006; Senthilkumar et al., 2009; Saikia et al., 2014). Such structures can be colonized by nitrogen-fixing bacteria like *Azorhizobium caulinodans* and *Azospirillum brasilense*, which favor nitrogen fixation compared to regular roots, and subsequently increase plant yield (Sriskandarajah et al., 1993; Kennedy et al., 1997; Christiansen-Weniger, 1998; Senthilkumar et al., 2009; Saikia et al., 2014). However, our knowledge of NLS formation is still fragmentary. For example, it is still not clear if the NLS formed in cereals are structurally similar to those formed on legume roots or what genes control NLS formation in cereals.

In this study, we established an experimental system in which NLS can be induced at a high frequency in rice and *M. truncatula*. We compared NLS formation in these plants at an anatomical level, and studied the regulation of NLS formation by genes involved in symbiosis in rice and *M. truncatula.* In order to identify genes and pathways involved in this process, we performed transcriptional profiling of NLS formation in rice by RNA-seq. We also investigated the expression profiles of a few select genes at different stages of NLS formation. Finally, we evaluated if NLS induced under these conditions could be colonized by nitrogen-fixing bacteria. This information provides a valuable resource for further exploration and understanding of the detailed molecular mechanisms underlying NLS development.

## Results

### In Response to Auxin, Rice and *M. truncatula* Roots Form Nodule-Like Structures in a Similar Fashion at a High Frequency

**FIGURE 1:**
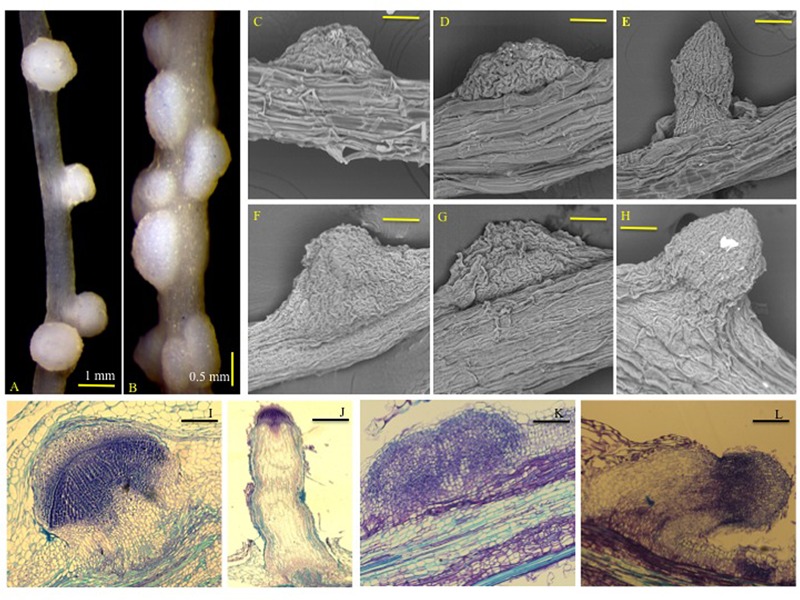
**Nodule-like structures (NLSs) appear to be structurally similar in rice and *Medicago* roots.** Images of **(A)** rice (Nipponbare cv.), and **(B)** *Medicago truncatula* (A17) NLS 14 dpt with 50 μM 2,4-D under controlled conditions. Images in **(A,B)** were taken at 12.5X magnification using a Leica MZ6 stereomicroscope. Images of rice (Nipponbare cv.) NLS at **(C)** 7 dpt and **(D)** 14 dpt captured by SEM. **(F,G)** Represent SEM images of *Medicago* (A17) NLS at 7 and 14 dpt, respectively. **(E,H)** Represent images of emerging lateral roots in rice and *Medicago*, respectively. SEM images were taken at 1400X magnification. Bars equal 80 μm. **(I,J)** Represent longitudinal ultra-thin sections of NLS and lateral root in rice. **(K,L)** Represent longitudinal ultra-thin sections of NLS and lateral root in *Medicago*. The sections are 5–8 μm in thickness.

We assayed formation of NLSs in rice and *M. truncatula* roots in response to the synthetic auxin, 2,4-Dichlorophenoxyacetic acid (2,4-D). 2,4-D was used in earlier studies to induce NLS in plant roots (Francisco and Akao, 1993; Ridge et al., 1993; Christiansen-Weniger, 1998). NLS formed along primary and lateral roots and were visible as early as 7 days post-treatment (dpt) with 2,4-D in both rice and *M. truncatula*. These structures had a broad base and were spherical at the distal end (**Figures 1A,B**). NLS appeared both independently and in clusters. Scanning electron microscopy (SEM) images showed that NLS did not change considerably in size between 7 and 14 dpt in both rice and *M. truncatula*. They appeared as spherical outgrowths with a smooth epidermis and lacked a clearly defined apical meristem (**Figures 1C,D,F,G**). The diffuse meristem was further confirmed by longitudinal ultra-thin sections (5 μm) of NLS in rice and *M. truncatula* (**Figures 1I,K**). Abundant vascular tissue differentiation was observed at the proximal and central portion of these structures. Lateral roots in rice and *Medicago* were easily distinguished from NLS by their narrow base, clearly defined apical meristem, and pericycle and apical meristem divisions (**Figures 1E,H,J,L**), in agreement with previous studies in sweet clover and *M. truncatula* (Wu et al., 1996; Rightmyer and Long, 2011).

**FIGURE 2:**
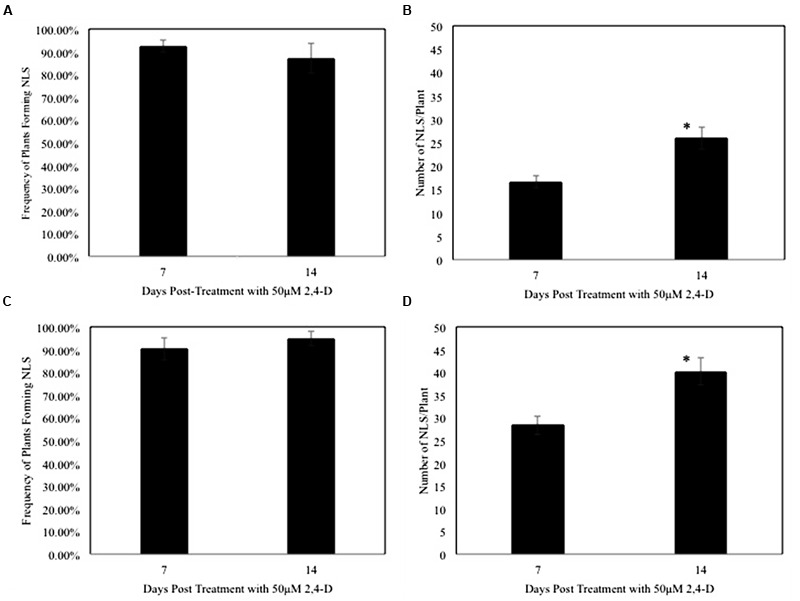
**Nodule-like structure frequency is high and the numbers increase over time in rice and *Medicago*. (A,C)** Show frequency of NLS formed per plant upon treatment with 50 μM 2,4-D at 7 and 14 dpt in rice and *Medicago* respectively. Data represents the average of five experimental replications (*n* = 10–15) ± SE. **(B,D)** Show that the average number of NLS increased significantly between 7 and 14 dpt in rice and *Medicago* roots respectively. Asterisk (^∗^) denotes significant difference between the two time points by *t*-test (*P* < 0.005). Data represents the average of five experimental replications (*n* = 10–15) ± SE.

Next we determined the efficiency of the auxin response by measuring the frequency of NLS formation over time. The frequency of NLS formation in rice was 92% at 7 dpt, and 87% at 14 dpt (**Figure 2A**). Similar to rice, we observed that NLS formed at a high frequency of 90% at 7 dpt and 95% at 14 dpt in *M. truncatula* (**Figure 2C**). Earlier studies using auxin transport inhibitors showed the frequency of NLS formation to be approximately 65% in sweet clover, and 38–83% in *M. truncatula* (Wu et al., 1996; Rightmyer and Long, 2011). Our results clearly indicate that 2,4-D can be successfully utilized to induce NLS formation in both rice and *M. truncatula* at a very high frequency. In addition to calculating frequency, we calculated the average number of NLS formed per plant. In rice, the average number of NLS formed per plant was 17 at 7 dpt and 26 at 14 dpt (**Figure 2B**). In *M. truncatula*, the average number of NLS formed per plant was 28 at 7 dpt and 40 at 14 dpt (**Figure 2D**). In both rice and *M. truncatula*, the average number of NLS formed at 14 dpt was significantly higher (Student’s *t*-test, *P* < 0.005) than at 7 dpt (**Figures 2B,D**). In a previous study with auxin transport inhibitors in *M. truncatula* the average number of NLS ranged between 1 and 8, 21 dpt (Rightmyer and Long, 2011). In past studies, ATIs were applied at a concentration of 50–100 μM to induce NLS in *M. truncatula* (Rightmyer and Long, 2011; Ng et al., 2015). In this study, we applied 2,4-D at a standard concentration of 50 μM for all our experiments. Besides NLS, we assessed other root phenotypes induced by the 2,4-D treatment. Our results show that lateral root formation was reduced in both rice and *M. truncatula* at 7 and 14 dpt (Student’s *t*-test, *P* < 0.005) (**Supplementary Figures S1A–D**). Besides that we did not observe any other phenotypic changes.

### NLS Formation in Symbiotic Mutants of Rice and *M. truncatula*

**FIGURE 3:**
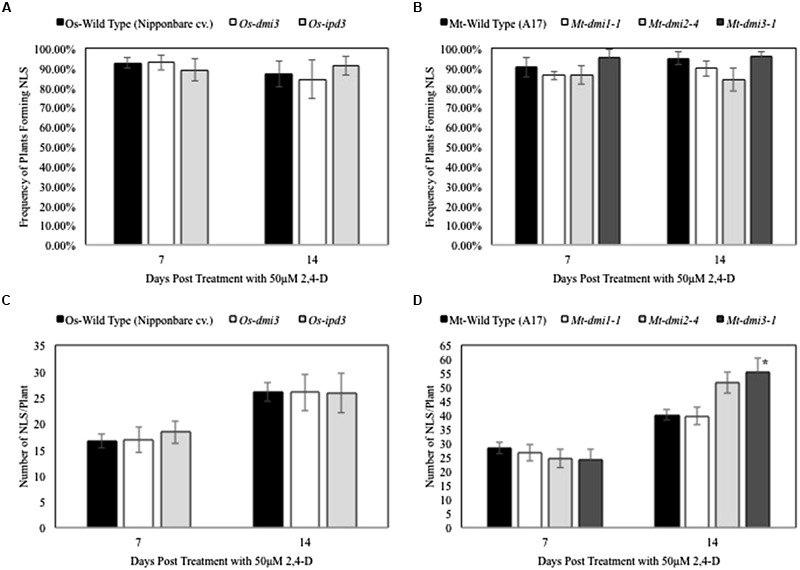
**Nodule-like structure formation in rice and *Medicago* is independent of the Common Symbiotic Pathway. (A,C)** Show that no significant differences were observed in NLS frequency in rice and *Medicago* CSP mutants and wild type plants at 7 and 14 dpt. Data represents average of at least three experimental replications (*n* = 10–15). **(B)** Show that the average number of NLS formed per plant in wild type rice and its symbiotic mutants was similar at both 7 and 14 dpt. Data represents average of at least three experimental replications (*n* = 10–15). **(D)** At 7 dpt, the average number of NLS formed in wildtype *Medicago* and its symbiotic mutants were similar. At 14 dpt, average NLS number in *dmi1* and *dmi2* mutants were similar to wild type, but *dmi3* formed significantly higher number of NLS than wild type. Asterisk denotes significant difference between *dmi3* and the other lines (one-way ANOVA, *F*_3,213_= 4.09, α = 0.05, *P* = 0.008; Dunnet’s *Post hoc*, wild type vs. *dmi3 P* = 0.035). Data represents the average of at least three experimental replications (*n* = 10–15) ± SE.

To determine if the CSP plays a role in 2,4-D-induced NLS formation, we compared NLS formation between wild type and symbiotic mutants in rice (*Os-dmi3* and *Os-ipd3*) and *M. truncatula* (*dmi1-1, dmi2-4*, and *dmi3-1)*. The frequency of NLS formation in *Os-dmi3* and *Os-ipd3* mutants was similar to wild type plants (*Oryza sativa* cv. Nipponbare) at both 7 and 14 dpt (**Figure 3A**). Similarly, the frequency of NLS formation in the tested *M. truncatula* CSP mutants (*dmi1-1, dmi2-4*, and *dmi3-1*) did not differ from the frequencies observed in the wild type (A17) plants at 7 and 14 dpt (**Figure 3C**). The average number of NLS formed in rice CSP mutants *Os-dmi3*, and *Os-ipd3* at 7 and 14 dpt was similar to the average number of NLS formed in the wild type line (**Figure 3B**). In *M. truncatula*, while the average number of NLS formed on *dmi1* and *dmi2* were similar to wild type at both 7 and 14 dpt, the average number of NLS formed on *dmi3* roots was significantly higher (one-way ANOVA, *F*_3,213_ = 4.09, α = 0.05, *P* = 0.008; Dunnet’s *Post hoc*, wild type vs. *dmi3 P* = 0.035) than wild type at 14 dpt (**Figure 3D**). In addition, we tested NLS formation in the *Medicago cre1* mutants. These mutants in the MtCRE1 cytokinin receptor are strongly affected in their ability to form nodules in presence of bacteria (Plet et al., 2011). While 2,4-D could induce NLS in *cre1-1* and *cre1-2*, the frequency of NLS formation in these mutants was lower (one-way ANOVA, *F*_2,90_ = 3.4511, α = 0.05, *P* < 0.001) than in wild type plants at 7 dpt (**Supplementary Figure S2A**). The average number of NLS in these mutants was also lower (Student’s *t*-test, *P* < 0.001) than the average number of NLS formed in the wild type plants (**Supplementary Figure S2B**).

### A Comprehensive Transcriptome Profile of Rice Roots Reveals Genes Involved in Response to Stimulus, Signaling, Anatomical Structure Development and Morphogenesis during NLS Formation

**FIGURE 4:**
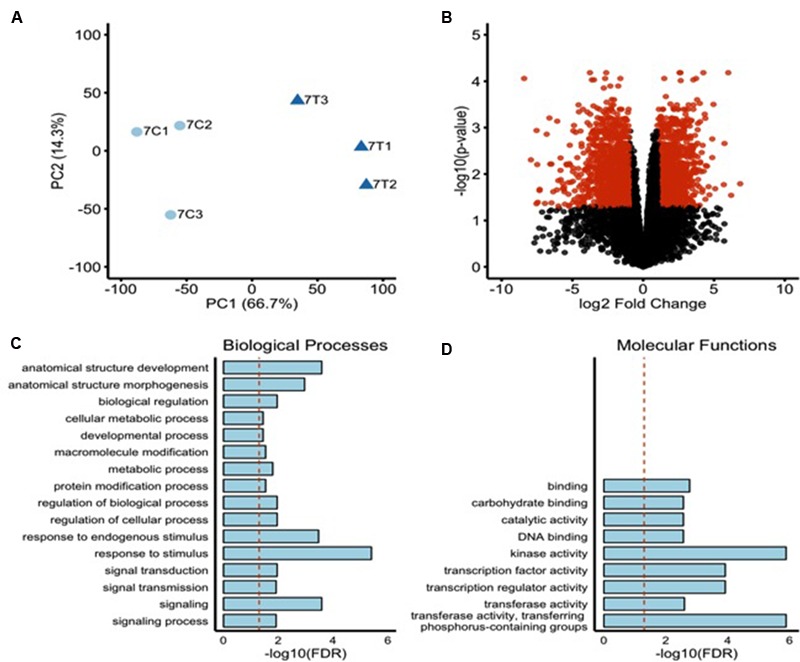
**Transcriptome profile of rice roots during NLS formation. (A)** Principal component analysis indicating a high level of similarity among biological replicates. Control samples = light blue circles, Treated sample = dark blue triangles. **(B)** Volcano plot showing FDR adjusted *P*-values and fold changes for all the genes with at least 10 counts per million in two or more samples. The mean log fold change is plotted vs. the -log10 of the differential expression FDR adjusted *P*-value. Points are colored red if their FDR adjusted *P*-value is <0.05 and their fold change is >2 (| LogFC| > 1). The upregulated DEGs were subjected to singular enrichment analysis (SEA) in agriGO gene ontology database using default parameters. **(C)** Bar chart of significantly enriched biological processes. Y-axis indicates the -log10 of the FDR adjusted *P*-value. Dotted red line indicates a FDR adjusted *P*-value = 0.05. **(D)** Bar chart of significantly enriched molecular functions. Y-axis indicates the -log10 of the FDR adjusted *P*-value. Dotted red line indicates a FDR adjusted *P*-value = 0.05.

To investigate the comprehensive gene expression profile of *O. sativa* cv. Nipponbare roots during 2,4-D-induced NLS formation, we analyzed the transcriptomes of rice roots containing NLS 7 dpt with auxin. Our treatment groups were (1) wild type roots + 2,4-D treatment and (2) wild type roots + mock treatment. Three biological replicates were included per treatment group. For treatment group 1, we used RNA samples only from plants that formed NLS. Illumina TruSeq libraries were prepared from these samples and 100 bp paired-end sequencing performed on an Illumina HiSeq2500. On average, 50 million reads were generated per sample with an average mapping rate to the *O. sativa* genome (MSU, version 7) of 94%. There was a high degree of correlation between the different biological replicates for each sample (**Figure 4A**). Over 15,000 transcripts were investigated in this RNA sequencing analysis. Differentially expressed genes (DEGs) were identified using a FDR adjusted *P*-value less than 0.05 and a twofold change cutoff (| Log_2_ (FC)|≥ 1) (**Figure 4B**). We identified 1991 DEGs, approximately 4% of total rice genes (Supplementary Table S1). Out of this subset, 991 genes were up-regulated in expression and 1000 genes were down-regulated in expression.

To investigate the biological significance of the genes regulated during NLS formation we assessed Gene Ontologies (GO) via singular enrichment analysis (SEA) with agriGO (Du et al., 2010). GO terms group genes by their biological processes, molecular functions, and the cellular location of their gene products. Twenty-five GO terms were significantly enriched among the 991 significantly upregulated genes. These included 16 biological processes (e.g., response to stimulus, signaling, signal transduction, anatomical structure development, anatomical structure morphogenesis) (**Figure 4C**) and nine molecular functions (e.g., kinase activity, transcription regulator activity, TF activity, and DNA binding activity) (**Figure 4D**). Genes involved in secondary metabolic processes, transport, and localization (biological processes) and transporter activity, catalytic activity, and lipid binding (molecular functions) were highly enriched among significantly downregulated genes (data not shown).

### Gene Expression Associated with Rice NLS Formation

For this study, we focused on four gene functional classes that are key components of signal transduction pathways: TFs, receptor kinases, transporters, and hormone-related genes. In the following sections, we report selected DEGs from these gene classes identified in our dataset.

#### Transcription Factors

We identified 170 DEGs belonging to several major TF gene families during auxin-induced NLS formation in rice roots (Supplementary Table S2). GO analysis indicated TFs involved in biological processes such as biosynthetic process, cellular metabolic process, response to endogenous stimulus, developmental process, multicellular organismal development, anatomical structure development and anatomical structure morphogenesis among others (**Supplementary Figure S3**). These TFs included Auxin response factors, APETALA2/Ethylene Responsive Factors (AP2/ERF), NAC domain containing proteins, basic helix-loop-helix (bHLH), Cysteine-2/Histidine-2 (C2H2) zinc finger, GAI-RGA-SCR (GRAS), lateral organ boundaries (LOB), and MYB-like protein families. Sixteen up-regulated genes and three down-regulated genes were characterized as AP2/ERF TFs, while nine up-regulated genes and eight down-regulated genes were NAC domain-containing TFs. Three auxin response factor TFs and two IAA-inducible TFs were upregulated in expression and four GRAS TFs were differentially regulated: three up-regulated genes and one down-regulated gene. WRKY TFs included eight up-regulated genes and two down-regulated genes and six up-regulated genes and 17 down-regulated genes were characterized as MYB TFs. Other genes in this category included basic helix-loop-helix, homeobox-leucine zipper family protein/ lipid-binding START domain containing proteins, WUSCHEL, and Lateral Organ Boundaries TF family members.

#### Protein Kinases

We identified 198 protein kinases in our analyses that were differentially regulated in expression during NLS formation (Supplementary Table S3). GO analysis indicated that a majority of these kinases are related to protein modification process, protein metabolic process, and signal transduction (**Supplementary Figure S4**). A large portion included cysteine-rich receptor like kinases (RLK), leucine-rich repeat RLKs, lectin protein kinases, and serine-threonine protein kinases. Among these were two up-regulated *BRI1-associated* receptor kinase genes, one up-regulated and one down-regulated *CLV1-like* LRR genes and one up-regulated gene *CLAVATA3/ESR (CLE)-related* kinase. Two up-regulated genes and two down-regulated genes were characterized as *CRINKLY4* kinase genes, four down-regulated genes were characterized as *STRUBBELIG* kinases, and two down-regulated genes were characterized as histidine kinases. Other receptors include one up-regulated abscisic acid (ABA) receptor genes and four up-regulated phytosulfokine receptor genes, as well as two up-regulated AGC kinases. Other receptor kinases differentially regulated in their expression included calcium-dependent protein kinases, CBL-interacting protein kinases, Kinase-interacting (KIP1-like) proteins, and several cysteine-rich RLKs and serine-threonine RLKs.

#### Transporters

We identified 191 DEGs involved in transport processes (Supplementary Table S4). Auxin eﬄux carriers and auxin influx carriers were identified with one up-regulated gene and one down-regulated gene in each class. A calcium-transporting ATPase was up-regulated and a cation eﬄux carrier and a sodium/potassium/calcium exchanger were downregulated. Additionally, two up-regulated genes and four down-regulated genes were characterized as ABC-2 type transporters and five up-regulated genes and 11 down-regulated genes were characterized as peptide transporters other than ABC-transporters. MATE eﬄux carriers were represented by seven up-regulated genes and four down-regulated genes. We also identified several nodulins belonging to distinct families such as *MtN21, MtN3, Nod26-like intrinsic proteins* (*NIP*), and *Major facilitator superfamily (MFS)*. We identified nine *MtN21* genes, three *MtN3* genes, three *NIP* genes, and seven *MFS* genes. Other transporters involved in transporting amino acid, ammonium, nitrate, potassium, copper, phosphate, glutamate, magnesium, sugar, sulfate, zinc, and chloride were also differentially expressed in NLS containing roots.

#### Hormone-Related

Besides those that are mentioned above, we identified several hormone-related genes (Supplementary Table S5). There were six up-regulated genes belonging to the auxin-responsive *GH3* family, one up-regulated auxin-responsive *SAUR* gene and four down-regulated *AIR12* genes. Two up-regulated genes encoded *Snakin/GASA* genes and three genes involved in jasmonate signaling were identified as up-regulated. Several genes involved in hormone biosynthesis were also differentially expressed. For instance, a *YUCCA* gene involved in auxin biosynthesis was up-regulated and two genes involved in ABA biosynthesis, three genes involved in gibberellin synthesis, and two genes involved in ethylene synthesis were up-regulated in their expression.

### Validation of RNA-Seq Data by RT-PCR

**FIGURE 5:**
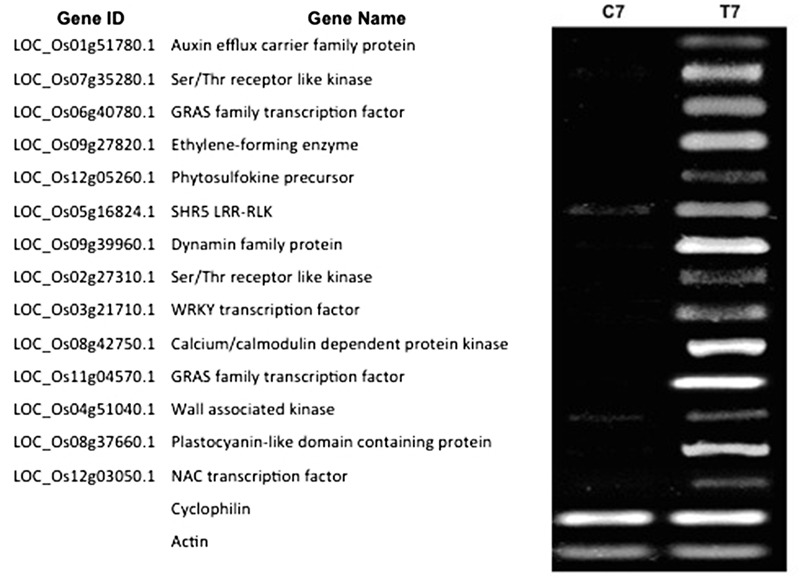
**Reverse transcription polymerase chain reaction (RT-PCR) validation of DEGs identified by RNA-Seq.** Expression pattern of 14 DEGs was validated by RT-PCR. For the RT-PCR experiments C7 and T7 represent cDNA templates synthesized from RNA samples at 7 dpt from control and treatment samples respectively. RT-PCR was performed with intron-spanning primers of the different genes in at least three biological replicates for all the samples. *Cyclophilin* and *Actin* were used as internal reference genes.

To confirm the validity of the gene expression patterns identified by the RNA-Seq data, the expression of 14 genes were examined by reverse transcription polymerase chain reaction (RT-PCR; **Figure 5**). We selected these genes based on their putative functions (e.g., TFs, protein kinases, transporters and hormone-related). RT-PCR primers were designed based on the Rice Genome Annotation Project database annotations, and their sequences are listed in Supplementary Table S6. Our RT-PCR results show that all the 14 genes examined were expressed at 7 dpt in NLS containing roots. This confirms the expression pattern of these genes identified via the RNAseq experiment.

### Gene Expression Patterns Vary during Different Stages of NLS Formation

**FIGURE 6:**
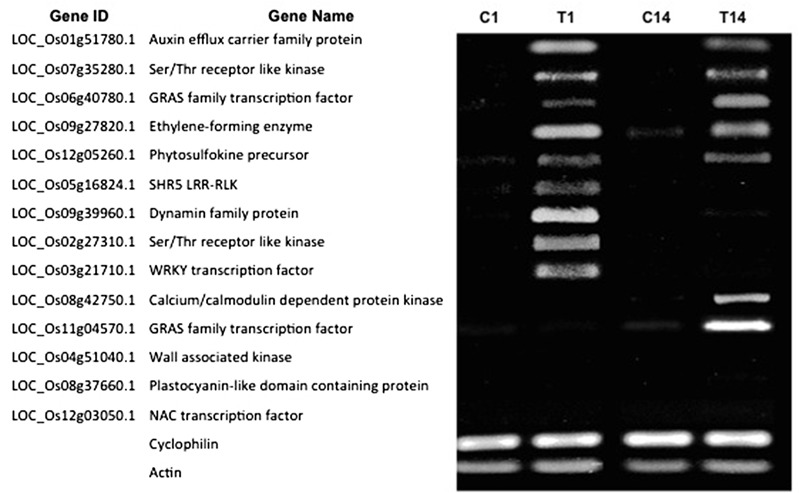
**Gene expression patterns vary during different stages of NLS formation.** Expression pattern of 14 DEGs at 1 and 14 dpt were checked by RT-PCR. For the RT-PCR experiments, C1 and T1, C14 and T14 represent cDNA templates synthesized from control and treatment RNA samples at 1 and 14 dpt respectively. RT-PCR was performed in at least three biological replicates for all the samples. *Cyclophilin* and *Actin* were used as internal reference genes.

We hypothesized that the DEGs at 7 dpt might exhibit differential expression patterns at early and/or later stages of NLS formation. Therefore, we investigated the expression pattern of the confirmed DEGs at two time points: 1 and 14 dpt. Our results show that five genes (Auxin eﬄux carrier family protein, Ser/Thr receptor like kinase, GRAS family TF, Ethylene-forming enzyme, and Phytosulfokine precursor) from this list were expressed at both 1 and 14 dpt (**Figure 6**). Interestingly, four genes (SHR5 Leucine-rich repeat transmembrane protein kinase, Dynamin family, Ser/Thr receptor like kinase, WRKY TF) were expressed at 1 dpt but not at 14 dpt (**Figure 6**). Similarly, we identified two genes (Calcium-dependent protein kinase and a GRAS family TF) that are expressed at 14 dpt but not at 1 dpt (**Figure 6**). Finally, we identified another group of genes (wall associated kinase, Plastocyanin-like domain containing protein, and NAC TF) that are not expressed at either 1 or 14 dpt but only at 7 dpt (**Figure 6**). Collectively, these results indicate that the plant utilizes different sets of genes at different stages of NLS formation.

### Nitrogen-Fixing Bacteria Can Colonize NLS on Rice Roots

**FIGURE 7:**
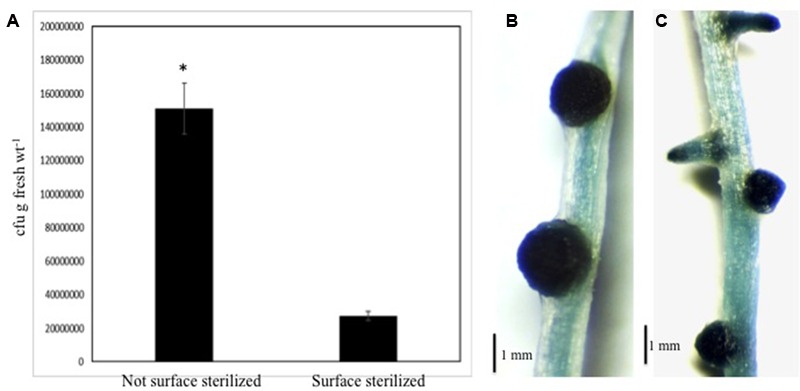
***Azorhizobium caulinodans* can colonize rice NLS. (A)** Comparison of total bacterial colony-forming units (cfu) determined by serial dilution and plate counts of bacteria between non-surface sterilized and surface sterilized (i.e., interior) roots of rice seedlings inoculated with *Azorhizobium caulinodans* ORS571 following treatment with 2,4-D. The data are the mean ± SE of five experiments. Each experiment had at least three plants. Asterisk (^∗^) denotes significant difference between the two conditions by *t*-test (*P* < 0.001). **(B,C)** Show localization of bacteria inside rice NLS and lateral roots by histochemical staining with X-Gal. The staining was performed following surface sterilization of these roots.

We wanted to determine if nitrogen-fixing bacteria could colonize NLS induced under our conditions. We selected *A. caulinodans* ORS571 (pXLGD4), carrying the *lacZ* reporter gene, for these studies (Gopalaswamy et al., 2000). Using plate count assays, we were able to recover *A. caulinodans* from surface sterilized rice roots containing NLS at 14 dpi (days post-inoculation). The number of colonies recovered from the surface sterilized roots was significantly lower (Student’s *t*-test, *P* < 0.001) than the non-surface sterilized roots (**Figure 7A**). Efficiency of surface sterilization was confirmed by lack of bacterial growth from last wash. These experiments suggested that bacteria could penetrate the NLS containing roots. Next we used histochemical staining with X-gal to determine if the bacteria were able to colonize NLS. Staining was performed on surface sterilized roots at 14 dpi. Following X-gal staining, a blue coloration indicating *lacZ* expression was observed throughout the NLS. This pattern was observed for all NLS in the roots indicating that NLS induced under our experimental conditions can be colonized by *A. caulinodans*. Besides NLS, the bacteria could colonize lateral roots as well (**Figures 7B,C**).

## Discussion

In this study, we established an efficient experimental system where NLS can be induced at a significantly higher frequency than previously reported in rice roots and the frequency of NLS formed does not change over time. We induced NLS formation in another land plant, the model legume, *M. truncatula*, under these same conditions. While earlier studies showed that auxin transport inhibitors (ATI) could induce NLS formation in legumes in general, we show that 2,4-D, a synthetic auxin, can also induce NLS formation specifically in *M. truncatula*. In fact 2,4-D induces NLS in *Medicago* roots at a high frequency similar to rice. The distribution, appearance, and when they start to appear are also similar in rice and *Medicago* and the number of NLS increased over time in both rice and *Medicago*. This suggests that the auxin perception and transport leading to NLS organogenesis is not a species-specific plant reaction and is likely conserved across land plants. To the best of our knowledge, previous studies investigating auxin-induced NLS in rice and other non-legumes were performed under non-controlled conditions in liquid medium (Francisco and Akao, 1993; Ridge et al., 1993; Sriskandarajah et al., 1993; El-Shahed and Abdel-Wahab, 2006; Narula et al., 2006). We adapted the experimental conditions used in a prior study in *M. truncatula* where NLS were induced under controlled aerobic conditions (Rightmyer and Long, 2011). To our knowledge, this is the first study that investigates NLS formation in a legume and a non-legume under identical experimental conditions. Based on these results, it seems this experimental system can be utilized to study NLS formation across land plants with similar sized root systems. Therefore, we propose the use of this efficient experimental system for future studies on NLS.

Like lateral roots, legume root nodules are also inducible lateral organs and these structures share several similarities and differences in their development (Desbrosses and Stougaard, 2011). Root nodules mainly originate from cortex cells although pericycle cells and the endodermis are also activated. Lateral root formation also starts with divisions in the pericycle, endodermis, and cortex cells, which is similar to nodule primordium initiation. However, at later stages in nodule primordia the frequency of divisions is highest in cortical layers, whereas in lateral root primordia mitotic activity is higher in pericycle. Also, root nodules have a peripheral vasculature while lateral roots have a central one (Desbrosses and Stougaard, 2011; Xiao et al., 2014). Since, NLS are also induced structures it is likely that there will be an overlap in the development of NLS with lateral roots and root nodules. Our results showed that lateral roots were easily distinguished from NLS by their narrow base, clearly defined apical meristem, and pericycle and apical meristem divisions. NLS were characterized by their broad base, spherical shape, diffuse meristem, and high cell differentiation in the vascular bundle. Within each NLS a single central vascular bundle is formed that is connected to the vascular system of the main root, which is similar to lateral roots. Although NLS originate from cortical cells similar to root nodules, they can be clearly distinguished by their diffuse meristem and cortical cell differentiation pattern. Nevertheless, NLS share some key features with both root nodules and lateral roots. Our results confirm past reports on NLS anatomy in sweet clover, *M. truncatula* and rice (Wu et al., 1996; Christiansen-Weniger, 1998; Rightmyer and Long, 2011). Also, the emergence of NLS through root cortex and epidermis make them comparable to nodules observed in the semiaquatic legume, *Sesbania rostrata* (Mathesius, 2008; Capoen et al., 2010). It is interesting to note that lateral root formation was reduced during NLS formation in both rice and *M. truncatula*. This suggests that NLS initiation might compete with lateral root emergence. Since this pattern is observed in both rice and *M. truncatula*, it is likely that this regulation of root architecture is conserved across land plants. However, further studies are required to determine what decides whether lateral roots or NLS will be initiated. Although, monocot and dicot roots differ in root architecture (Hochholdinger et al., 2004), we did not observe any significant differences in NLS development in rice and *Medicago*. While further studies are required to offer more insights into their similarities and differences, our results strongly suggest that NLS formation follows a common developmental program in land plants. It is therefore, likely that there will be a conservation of the genetic pathway leading to NLS formation across land plants.

In order to understand the genetic regulation of NLS in rice and *Medicago*, we studied NLS formation in plant mutants affected in the CSP. Our results show that the symbiotic mutants in rice (*dmi3* and *ipd3*) and *Medicago* (*dmi1, dmi2*, and *dmi3*) retain the ability to form NLS in response to the auxin treatment. This suggests that the action of auxin leading to cell divisions and morphogenesis occurs downstream of these early symbiotic signaling genes including CCaMK. These results are consistent with a prior study in *Medicago* where it was shown that ATI-induced NLS also do not require CCaMK (Rightmyer and Long, 2011). In addition, we show that this mechanism is conserved in rice further supporting the concept of a shared developmental program in land plants.

Spontaneous nodule formation is also mediated by a gain-of-function mutation of *LHK1/CRE1*, which encodes a putative cytokinin receptor (Tirichine et al., 2007). The formation of NLS in nodulation defective mutants (e.g., *nsp2* and *nin*) acting downstream of *CRE1* suggest that the auxin transport control of nodule organogenesis acts downstream of cytokinin signaling (Rightmyer and Long, 2011). In addition, application of cytokinins induces auxin responses during NLS formation in white clover (Mathesius et al., 2000) and auxin responses are also detected during NLS formation in *Lotus japonicus snf2* mutant (Suzaki et al., 2012). Here, we show that NLS formation can be induced in roots of *cre1-1* and *cre1-2* mutants by 2,4-D application. This supports findings from a previous study in *Medicago* where ATI could induce NLS in *cre1-1* mutants (Ng et al., 2015). Collectively, these results are consistent with a model in which NLS formation occurs downstream of cytokinin-induced step(s) (Rightmyer and Long, 2011; Ng et al., 2015). Whether this model is conserved in non-legumes will need further investigations.

In recent years, RNA-seq has been used as a powerful and cost-efficient tool to profile transcriptomic changes. Our genome-wide RNA-seq analysis of rice roots identified 100s of up-regulated and down-regulated genes in NLS containing roots compared to mock treated roots. Our results suggest that a vast array of genes encoding TFs, protein kinases and transporters are mediating the plant’s response to auxin and its transport leading to NLS organogenesis. It is expected that the genetic pathway controlling NLS will share some common elements with the pathways leading to lateral root development and root nodule organogenesis. In our dataset we identified several promising candidate genes that have been implicated in root development. In the following sections we discuss selected genes, which are likely to be involved in NLS development.

Transcription factors will play key roles in triggering the NLS developmental program that will involve many other genes. TFs related to response to endogenous stimulus, developmental process, anatomical structure development and anatomical structure morphogenesis were enriched in our dataset. The APETALA2/Ethylene Responsive Factors (AP2/ERF) is a large family of TFs that play a vital role on plant growth and enables plants to respond to environmental stimuli. We identified numerous DEGs (e.g., LOC_Os05g29810.1, LOC_Os09g39850.1, LOC_Os03g64260.1, LOC_Os11g19060.1, LOC_Os02g40070.1 etc.) belonging to this family majority of which were upregulated in their expression. Interestingly, we identified a *PLETHORA2* gene (LOC_Os02g40070.1) encoding AP2 class TFs, which is essential for quiescent center (QC) specification and stem cell niche in the root meristem (Aida et al., 2004; Benjamins and Scheres, 2008). The *PLT* genes are transcribed in response to auxin accumulation and are dependent on auxin response TFs. *PLT* genes are also necessary for the expression of *PIN* genes to mediate auxin distribution suggesting a possible role in NLS formation (Aida et al., 2004; Benjamins and Scheres, 2008). Another category well-represented in our dataset was the NAC TFs (e.g., LOC_Os07g37920.1, LOC_Os01g60020.1, LOC_Os02g15340.1, LOC_Os11g03370.1, etc.). These TFs have been implicated in root development and auxin signaling (Nuruzzaman et al., 2013). In *Arabidopsis, NAC1* has been shown to mediate auxin signaling to promote lateral root development (Xie et al., 2000). Interestingly, NAC TFs have only been identified in plants with approximately 150 genes in rice (Nuruzzaman et al., 2013). Whether these are required for NLS development will require further studies. The next important class of TFs in our dataset was the GRAS family TFs. We identified four GRAS family TFs (e.g., LOC_Os06g40780.1, LOC_Os03g51330.1, LOC_Os11g04570.1, LOC_Os07g40020.1) that were differentially expressed in NLS containing roots. GRAS proteins are an ancient family of plant-specific TFs that play diverse roles including shoot and root development (Hirsch and Oldroyd, 2009). More importantly GRAS family TFs, NSP1 and NSP2, are required for nodule organogenesis (Kalo et al., 2005; Smit et al., 2005; Heckmann et al., 2006). It is therefore tempting to speculate the involvement of GRAS proteins in NLS development. Interestingly, we identified several *Lateral Organ Boundaries Domain family* (e.g., LOC_Os02g57490.1, LOC_Os08g44940.1, LOC_Os10g07510.1, etc.) genes whose expressions were increased during NLS formation. *LBD* genes are critical for root development in both dicots and monocots and are directly involved in auxin signal cascades regulating root development (Liu et al., 2005; Okushima et al., 2007; Lee et al., 2009). One recent study showed that *AUX1* and *LAX3* auxin influx carriers are required for auxin signaling that activates *LBD16* and *LBD18* to control lateral root development in *Arabidopsis* (Lee et al., 2015). Future studies will focus on functionally characterizing these promising candidates and identifying their target genes.

The phytohormones auxin, cytokinin, brassinosteroids, and ABA play key roles in regulating plant growth in response to internal and external stimuli (Vanstraelen and Benkova, 2012). For instance, the interaction between auxin and cytokinin is especially significant to control the formation and maintenance of meristems (Su et al., 2011). This interplay between auxin and cytokinin is also important for lateral root and nodule formation (Mathesius, 2008; Ding and Oldroyd, 2009; Desbrosses and Stougaard, 2011; Mukherjee and Ané, 2011b; Ryu et al., 2012; Bensmihen, 2015). Altering the auxin to cytokinin balance specifies whether root cells divide in the pericycle or in the cortex (Mathesius, 2008). We identified two histidine kinase receptors (LOC_Os03g50860.1 and LOC_Os12g26940.1) that were both down-regulated in expression during NLS formation. Constitutive activity of the cytokinin receptor has already been reported to be sufficient to initiate NLS in the absence of rhizobia (Gonzalez-Rizzo et al., 2006; Murray et al., 2007; Tirichine et al., 2007). It needs to be seen whether a crosstalk between cytokinin and auxin is involved in auxin-induced NLS development. Brassinosteroids (BRs) also interact with auxin in regulating plant development. For example, both BRs and auxin pathways synergistically regulate the expression of several auxin-responsive genes and regulate root development (Mussig et al., 2003; Mouchel et al., 2006; Clouse, 2011). The RLK coreceptor BRI-Associated Kinase-1 (BAK1) partners with multiple ligand-binding RLKs and is involved in Brassinosteroids (BRs)-mediated plant growth and development via auxin regulation (Clouse, 2011; Kim et al., 2013). We identified *BRI1-associated receptor kinase 1* (LOC_Os11g31540.1 and LOC_Os05g34270.1) genes, which are upregulated in their expression in NLS containing roots and are excellent candidate genes. Another class of peptide hormones, phytosulfokines, has been suggested to play a role in root development (Kutschmar et al., 2009; Yamada and Sawa, 2013). We identified several phytosulfokine receptors (LOC_Os02g05970.1, LOC_Os06g47760.1, LOC_Os04g57630.1, and LOC_Os07g01710.1) to be differentially expressed during NLS formation. PSK receptor proteins share great structural similarity to BRI1 and in fact require BR to regulate growth (Ladwig et al., 2015). It will be interesting to determine if these genes are functionally involved in NLS formation. Although, the roles of ABA and auxin in plant growth are distinct, sensitivity to ABA correlates with sensitivity to auxin (Vanstraelen and Benkova, 2012; Thole et al., 2014). We identified an ABA receptor (LOC_Os05g12260.1) to be upregulated in its expression during NLS formation. It is possible that a crosstalk between ABA and auxin mediates NLS development.

Plants have developed intricate signaling systems that employ secreted peptides and membrane bound receptor kinases for short- and long-range communication (Stahl and Simon, 2012; Yamada and Sawa, 2013). Peptides belonging to the CLV3/EMBRYO SURROUNDING REGION (CLE) family act as signals in several of these pathways including root development and nodule formation (Stahl and Simon, 2012; Yamada and Sawa, 2013). In rice, FON2, closely related to CLV3, regulates meristem size and requires FON1, an ortholog of CLV1 (Suzaki et al., 2006). Importantly, the CLV1 ortholog (SUNN) in legumes plays a key role in regulating nodule numbers (Schnabel et al., 2005). In addition, two CLE genes of *M. truncatula, MtCLE12* and *MtCLE13*, have been shown to play a role in controlling nodule number (Mortier et al., 2012). We identified FON2 SPARE1 (FOS1; LOC_Os02g21890.1) and CLV1-like LRR (LOC_Os07g04190.1, LOC_Os06g50340.1), which are differentially expressed during NLS formation. In addition, we identified *CRINKLY4* (LOC_Os11g44430.1, LOC_Os11g44500.1, LOC_Os04g35890.1, LOC_Os11g11490.1) in our list of DEGs. Studies on root development and the distal root meristem have revealed a role for CRINKLY4 (ACR4) (De Smet et al., 2008; Stahl and Simon, 2012). It will require further investigation to determine if the peptides and their receptors are involved in NLS development. Several RLKs are known to be important for the control of organ size and shape. Well-characterized examples include the brassinosteroid hormone receptor (BRI1), stem cell regulator CLV1, and CRINKLY4, which are involved in epidermal differentiation and formative cell division control in the root pericycle. *STRUBBELIG* (*SUB*) is another LRR-RLK gene with a role in tissue morphogenesis of several plant organs (Chevalier et al., 2005; Vaddepalli et al., 2011). In our analysis, we identified several *SUB* (e.g., LOC_Os05g25540.1, LOC_Os02g04430.1, LOC_Os10g25090.1, LOC_Os06g42800.1) genes and all of these RLKs are downregulated in their expression. Whether reduction of *SUB* transcript levels correlate with NLS size and shape needs further investigation. Plant AGC kinases are an exciting group of kinases that have been shown to perform plant-specific functions. Interestingly, some of these have been shown to be involved in modulation of auxin responses and the regulation of auxin transport (Robert and Offringa, 2008). Studies in *Arabidopsis* have shown that the *PIN* auxin eﬄux carriers are one of the main targets of these kinases (Robert and Offringa, 2008). AGC kinase also acts on an auxin transporter of the *ABCB* family, besides phosphorylating *PIN* auxin eﬄux carriers (Rademacher and Offringa, 2012). Furthermore, an *IRE-like AGC* kinase gene, *MtIRE*, has been shown to play a role during legume–rhizobia symbiosis (Pislariu and Dickstein, 2007). We identified two AGC kinases (LOC_Os03g44020.1 and LOC_Os11g05320.1) that are up-regulated in expression in NLS containing roots. Additional investigations are required to prove if these genes are directly involved in NLS formation.

We identified 191 transporters in our list of DEGs. Several peptide transporters including the ABC-2 type transporters (e.g., LOC_Os04g11820.1, LOC_Os08g30780.1, LOC_Os05g02890.1, etc.) whose expression was differentially regulated were identified. In plants, these proteins have been shown to be involved in multiple processes including transport of auxin (Geiser and Murphy, 2005). Since auxin transport is an integral part of the NLS organogenesis process, these are promising candidates. Other genes involved in auxin transport, eﬄux and influx, were also differentially regulated in expression. While the auxin eﬄux carrier *OsPIN8* (LOC_Os01g51780.1) was upregulated in its expression, the auxin eﬄux carrier *OsPIN5c* (LOC_Os09g32770.1) was downregulated in expression. Similarly, auxin influx carrier *OsLAX1* (LOC_Os03g14080.1) was upregulated in its expression and the auxin influx carrier *OsLAX3* (LOC_Os11g06820.1) was downregulated in expression. Several genetic and biochemical evidence have shown that these eﬄux and influx carriers mediate auxin-related developmental programs in different plant organs and tissues and influence root development (Swarup and Peret, 2012; Adamowski and Friml, 2015). These auxin transporters also play a role in root nodule formation (Huo et al., 2006). In addition, synthetic auxins like 2,4-D and napthalene1-acetic acid (NAA) are transported specifically by either auxin influx or eﬄux carriers (Peer et al., 2011; Hosek et al., 2012). Since, we used 2,4-D to induce NLS in rice roots, the identification of these auxin transporters is not unexpected. Therefore, it will not be surprising for these genes to play a role in 2,4-D-induced NLS formation. However, since ATIs (e.g., NPA and TIBA) are known inhibitors of auxin eﬄux carriers, the role of these auxin transporters might be different in ATI-induced NLS (Peer et al., 2011; Hosek et al., 2012). Our dataset revealed differential expression of several *MATE* eﬄux family genes (e.g., LOC_Os10g20450.1, LOC_Os07g31884.1, LOC_Os04g30490.1, LOC_Os08g43654.1, etc.). This recently characterized transporter family has been implicated in plant growth and development. We also observed differential expression of several nodulin-like genes corresponding to distinct families: *MtN21* (e.g., LOC_Os12g33300.1, LOC_Os02g52930.1, LOC_Os06g01660.1, etc.), *MtN3* (LOC_Os01g36070.1, LOC_Os01g50460.1, and LOC_Os01g12130.1), *NOD-26-like intrinsic proteins* (LOC_Os02g13870.1, LOC_Os02g51110.1, and LOC_Os06g12310.1), and *Major Facilitator Superfamily* (e.g., LOC_Os03g11900.1, LOC_Os04g42130.1, LOC_Os07g03910.1, etc.). Nodulin-encoding genes were first defined as legume genes that are specifically expressed during legume–rhizobia symbioses (Oldroyd and Downie, 2008; Mukherjee and Ané, 2011b). Interestingly, homologs of nodulin genes have been identified in several non-legumes suggesting a possible role of these genes in plant physiology (Denance et al., 2014). Some of these genes have been shown to be involved in transporting several solutes such as nutrients, amino acids, and hormones throughout plant development (Denance et al., 2014). Future studies will shed light on how these nodulins in non-legumes are involved in plant development and other processes.

We identified several hormone-related genes many of which are related to auxin response or hormone biosynthesis. For instance, several DEGs belonging to the auxin responsive *GH3* gene family (e.g., LOC_Os05g42150.1, LOC_Os01g55940.1, LOC_Os07g40290.1, LOC_Os11g32520.1, LOC_Os11g32510.1) were upregulated in their expression. The rice *GH3* gene family includes 13 paralogs, four belonging to group I (*GH3-3, -5, -6*, and *-12*) and nine belonging to group II (*GH3-1, -2, -4, -7, -8, -9, -10, -11*, and *-13*) (Fu et al., 2011). Interestingly, we detected *GH3* genes belonging to only group II in this study. Several *Arabidopsis* and rice *GH3* group II paralogs have been identified to have influence on root development (Fu et al., 2011). Therefore, these genes are also excellent candidates. We identified a *YUCCA* (*LOC_Os04g03980.1*) gene involved in auxin biosynthesis. The *YUCCA* gene encodes a flavin monooxygenase and is involved in Trp-dependent auxin biosynthesis. Genetic studies have shown that *yucca* mutants do not develop a root meristem (Benjamins and Scheres, 2008). It is likely that the *YUCCA* overexpression leading to increased auxin biosynthesis is a critical factor for NLS formation. We also identified two *Snakin/GASA* (LOC_Os03g41060.1 and LOC_Os03g55290.1) genes that are upregulated in their expression. Most of the *Snakin/GASA* genes are regulated by plant hormones and are involved in hormonal signaling pathways modulating hormonal levels and responses (Nahirnak et al., 2012). Importantly, Snakin/GASA proteins have been shown to be involved in plant development and plant environmental responses (Nahirnak et al., 2012). Based on these facts these are promising candidates for further studies.

It needs to be added that none of the genes from the CSP, *OsDMI3* (LOC_Os05g41090.1), *OsIPD3* (LOC_Os06g02520.1), *OsCASTOR* (LOC_Os03g62650.1), or *OsPOLLUX* (LOC_Os01g64980.1), were differentially expressed in NLS containing roots. This supports our earlier results about the role of this pathway during NLS formation. Another interesting observation was the lack of any auxin receptors (ex. *TIR1*) in our dataset. While we detected several components of the auxin signaling components (e.g., *AUX/IAA, ARF, PLT*, etc.), auxin transport components (e.g., *PIN, MDR* and *PGP, AUX1*) and even auxin biosynthesis (*YUCCA*), the auxin receptor class was missing. It is likely that the auxin receptors act very early during NLS organogenesis.

Overall, this data provides comprehensive information of gene expression during NLS formation in rice, facilitating our understanding of the molecular events involved in this process. In this study, we also show that the expression pattern of some of these genes vary over time. For instance, some genes (auxin eﬄux carrier LOC_Os01g51780.1, Ser/Thr RLK LOC_Os07g35280.1, GRAS family TF LOC_Os06g40780.1, phytosulfokine precursor LOC_Os12g05260.1, and ethylene forming enzyme LOC_Os09g27820.1) are expressed at 1, 7, and 14 dpt suggesting a possible role from the early hormone perception to the later stages of NLS development and maintenance. Similarly, some genes (Dynamin family protein LOC_Os09g39960.1, SHR5 LRR-RLK LOC_Os05g16824.1, Ser/Thr RLK LOC_Os02g27310.1, and WRKY TF LOC_Os03g21710.1) are expressed at 1 and 7 dpt but not at 14 dpt indicating their participation in the early stages leading to NLS formation. Another set of genes (calcium/calmodulin dependent protein kinase LOC_Os08g42750.1, and GRAS family TF LOC_Os11g04570.1) were differentially expressed at 7 and 14 dpt suggesting a possible role in the later stages involving NLS organogenesis. All of these data taken together clearly indicate that the plant uses different sets of genes during the different stages of NLS organogenesis.

Finally, we were interested to determine if NLS induced on rice roots under our conditions could be colonized by nitrogen-fixing bacteria. We used *A. caulinodans* in these experiments as it is a known nitrogen-fixer and can colonize rice roots (Gopalaswamy et al., 2000; Dixon and Kahn, 2004). Using plate count assays we first show that *Azorhizobium* can penetrate plant roots containing NLS under our conditions. This suggests that the 2,4-D treatment does not inhibit *Azorhizobium* to penetrate the rice roots under these conditions. Expectedly the number of bacterial colonies recovered post-surface sterilization was significantly fewer than when the roots were not surface sterilized. Next using histochemical staining we show that rice NLS can be colonized by *A. caulinodans*. The *lacZ* gene expression was observed throughout NLS suggesting that the central meristem of the NLS might be a major colonization niche for *A. caulinodans*. The NLS meristem might be a preferential site for bacterial colonization due to their rapid and uncontrolled growth. Interestingly, this pattern was detected on NLS throughout the root system suggesting that bacterial colonization might be similar for all NLS irrespective of their location on the root. In addition to NLS, bacteria also colonized lateral roots. Past studies have shown that bacteria can infect lateral roots via a ‘crack entry’ mechanism (Reddy et al., 1997). The emergence of NLS through the intact root cortex and epidermis suggest that bacteria might use a similar mechanism to penetrate NLS. Clearly further studies are required to elucidate the exact mechanism of colonization. It also needs to be determined if colonization by *A. caulinodans* is a specific event or whether introduced bacteria generally colonize these structures. Further experimentation is required to determine if other nitrogen-fixing bacteria can colonize NLS and if their colonization patterns overlap. Nevertheless our results show that NLS induced on rice roots under our experimental conditions can be colonized by bacteria.

## Conclusion

Studies in legume–rhizobia symbiosis have shown that cortical cell divisions leading to nodule organogenesis can be triggered independent of bacteria. Plant hormones play a critical role in inducing the development of these NLSs. Moreover, NLS can be induced in both legumes and non-legumes. This offers a unique opportunity to study hormone-induced root organogenesis not just in legumes but also in non-legumes, especially cereals. We established an experimental system that can induce NLS consistently at a high frequency in plant roots. Our results suggest that the developmental program is conserved in land plants. However, more studies are required to offer further insights into the development of NLS. We also performed a RNA-seq experiment to profile the transcriptomic responses in rice roots that occur during NLS formation via 2,4-D treatment. We identified several genes related to response to stimulus, signaling, anatomical structure development, and anatomical structure morphogenesis. These will serve as excellent candidates for future studies to genetically characterize the pathway leading to NLS formation in rice. It will be interesting to identify rice genes differentially expressed in roots where NLS are induced by ATIs. It is likely there will be some key genes that are commonly expressed by both treatments. It is also not unreasonable to identify some genes that are specific to each treatment. Future studies can also focus on identifying commonly expressed genes and the pathways during NLS formation in other land plants such as *M. truncatula* and *B. distachyon*. The power of comparative genomics can be exploited to compare and contrast the genetic pathways leading to NLS formation across different land plants. Identification of the genetic switches controlling this nodule-like organogenesis program in non-legumes, especially cereals will have tremendous biotechnological implications. Finally, we show that NLS in rice could be colonized by *A. caulinodans.* Taken together all these results suggest that this experimental system can be used for future studies on NLS in grasses.

## Materials and Methods

### Plant Material and Growth Conditions

For studies in rice, we used *O. sativa* cv. Nipponbare and *Tos17* insertion lines in *DMI3* (line NF8513), and in *IPD3* (line NC0263) for NLS studies and RNA-seq experiments (Chen et al., 2007, 2008). The seeds of the above-mentioned rice genotypes were surface-sterilized in 2% (v/v) sodium hypochlorite solution for 30 min, rinsed five times with distilled water and then allowed to imbibe in the distilled water for 24 h as described in Mukherjee and Ané (2011a). Following the 24-h period, the seeds were transferred under sterile conditions to 9-cm Petri plates (#633185, Greiner bio-one, Monroe, NC, USA) lined with germination paper (Anchor Paper, Saint Paul, MN, USA). Plates were sealed with parafilm (L-2020-1, BioExpress, Kaysville, UT, USA), wrapped completely in aluminum foil, and then allowed to germinate at room temperature (25°C) for approximately 72–96 h. Germinated seedlings were transferred to 15-cm Petri plates (#639102, Greiner bio-one, Monroe, NC, USA) containing low-N_2_ Fahraeus medium and allowed to grow for 7 days in a Percival growth chamber (#CU-22L, Perry, IA, USA) with a 16-h, 22°C day and 8-h, 24°C night cycle and 150–200 μmol m^-2^s^-1^ light intensity, and relative humidity of 65% prior to hormone treatment.

For studies in *M. truncatula*, we used *M. truncatula* Jemalong A17, *dmi1-1, dmi2-4, dmi3-1, cre1-1*, and *cre1-2* (Catoira et al., 2000; Ané et al., 2004; Plet et al., 2011). Seeds of the above-mentioned *M. truncatula* genotypes were surface sterilized in concentrated sulfuric acid for 6 min. Seeds were rinsed five times with distilled water followed by one rinse with 2.4% (v/v) sodium hypochlorite solution. Seeds were rinsed an additional five times with distilled water and then allowed to imbibe for a minimum of 2 h in a sterile environment. After the imbibition period, sterilized seeds were transferred to 9-cm Petri plates (#633185, Greiner bio-one, Monroe, NC, USA) containing 1% (w/v) plant agar (#A038-2.5KG, Caisson Laboratories, Inc., Smithfield, UT, USA) supplemented with 1 mM Gibberellic acid (GA3) medium under sterile conditions. The plates were wrapped completely in aluminum foil and placed at 4°C for a minimum of 48 h. Plates were placed at room temperature (25°C) for an additional 48 h to allow the seeds to germinate. Germinated seedlings were transferred to 15-cm Petri plates (#639102, Greiner bio-one, Monroe, NC, USA) containing low-N_2_ Fahraeus medium and allowed to grow for 7 days in a Percival growth chamber (#CU-22L, Perry, IA, USA) with a 16-h, 22°C day and 8-h, 24°C night cycle and 150–200 μmol m^-2^s^-1^ light intensity, and relative humidity of 65% prior to hormone treatment.

### Hormonal Treatment and Plant Phenotype Scoring

For hormone treatments of rice and *Medicago* seedlings, we used the synthetic auxin, 2,4-Dichlorophenoxyacetic acid (2,4-D) (CAS# 94-75-7, Caisson Laboratories, Inc., Smithfield, UT, USA). Seven-day-old whole plants of rice and *Medicago*, grown as mentioned above, were soaked in 50 μM of 2,4-D solution for 1 h under sterile conditions. Ten ml of 2,4-D solution was used per plant for treatment solution. Following this treatment, the plants were transferred to 15-cm Petri plates (#639102, Greiner bio-one, Monroe, NC, USA) containing low-N_2_ Fahraeus medium. All plants were grown in a Percival growth chamber (Model #: CU-22L, Perry, IA, USA) with a 16-h, 22°C day and 8-h, 24°C night cycle and 150–200 μmol m^-2^s^-1^ light intensity at 65% relative humidity. Plants were phenotypically scored for NLS formation after 7 and 14 days post-treatment. Non-treated (mock) rice and *Medicago* plants were cultivated as described above except 2,4-D was not included in the treatment solution to be used as controls for our studies.

The NLS phenotype was scored using a Leica MZ6 stereomicroscope (Leica Microsystems, Inc., Buffalo Grove, IL, USA) to visualize the NLS at 12.5X magnification on all rice and *M. truncatula* roots. The frequency of NLS formation was calculated as the number of plants forming NLS out of the total number of plants treated with 2,4-D. Statistical analysis of scored phenotypic data, frequency and average number of NLS, was analyzed using JMP^®^ 11 (SAS Institute, Inc., Cary, NC, USA). Log_10_ transformation of data was used to meet the assumptions of parametric testing.

### Bacterial Inoculation, Bacterial Counts, and X-gal Staining

The bacterial inoculation of rice roots was performed as described by Mitra et al. (2016). Briefly, following hormone treatment as mentioned earlier rice seedlings were inoculated with or without the *lacZ*-marked *A. caulinodans* ORS571 (pXLGD4). Bacteria were grown on LB/Tetracycline (10 μg/ml) plates at 28°C until they reached an optical density of 0.6. The seedlings were inoculated with 10^8^ cells/ml with *A. caulinodans*. Following inoculation, all plants were grown in the growth chamber as mentioned earlier.

The colonization was quantified as described by Dong et al. (2003) and Mitra et al. (2016). Briefly, seedlings were sampled 14 days post-inoculation and loosely attached bacteria were removed by washing the roots in sterile water. Surface sterilization was performed by immersing the plant roots in sterilization solution (1X PBS, 1% bleach, 0.1% sodium dodecyl sulfate, 0.2% Tween 20) for at least 1 min followed by three washes with sterile water. The samples were then crushed manually using sterile mortar and pestle. The homogenates were resuspended in 1 ml PBS containing 20% glycerol. This suspension was serially diluted in 10-fold increments and cultured on LB plates supplemented with tetracycline (10 μg/ml). To determine the efficacy of surface sterilization, the wash from the last root rinse was cultured on LB/tetracycline plates. Lack of bacterial growth from the last wash indicated efficiency of surface sterilization.

Bacterial colonization of the roots of inoculated plants was visualized as described by Gopalaswamy et al. (2000). Briefly, the bacteria were visualized by light microscopy of the blue precipitate resulting from the degradation of 5-bromo-4-chloro-3-indolyl-β-galactopyranoside (X-Gal) by β-galactosidase. Seedlings were fixed in 2% (v/v) glutaraldehyde in 1X PBS for 30 min. The plants were next washed three times with 1X PBS solution before incubation with X-Gal for 1 h. Prior to staining, all seedlings were thoroughly surface sterilized as mentioned earlier.

### Scanning Electron Microscopy

Scanning electron microscopy was performed as previously described by Umali-Garcia et al. (1980) with the following exceptions: roots containing NLS were dehydrated in 70% ethanol and stored at 4°C prior to preparation for SEM analysis. Samples of NLS were cut into 1–2 mm sections and dried to a critical point (Pelco CPD2 Critical Point Dryer, Ted Pella, Inc., Redding, CA, USA) (Umali-Garcia et al., 1980). Sections were then coated with ∼10 nm gold layer in a Denton Vacuum Desk IV sputter coater (Denton Vacuum, LLC, Moorestown, NJ, USA) for SEM preparation. NLS were examined using a P-SEM 2000 scanning electron microscope (ASPEX Corporation, Delmont, PA, USA) at 10–12 keV (Umali-Garcia et al., 1980).

### Ultra-Thin Sectioning

Plant tissues were fixed in ethanol (70% v/v) for 3 days and then transferred to phosphate buffer. Tissues were processed for paraffin embedding using an automated tissue processor. Processing included ethanol dehydration, clearing in xylenes, and vacuum infiltration with paraffin. Sections were cut on a microtome (Shandon Finesse 325 Manual Microtome, Thermo Fisher) at a thickness of 5 μm. The slides were stained with toluidine blue. The sectioned root tissues were later screened for NLS and lateral root sections using an Olympus CX21 microscope.

### RNA Extraction

For this study, total RNA was extracted from untreated plant roots and 50 μM 2,4-D treated plant roots at 1, 7, and 14 days post-treatment (dpt). Specifically for treatment samples at 7 and 14 dpt, RNA was extracted only from plant roots containing NLS. Total RNA was extracted using Qiagen RNeasy^®^ Plant Mini Kit (Cat #74904, Foster City, CA, USA) per manufacturer’s protocol. We included three biological replicates for each sample.

### RNA Sequencing

RNA sequencing including RNA quantification and library preparation was performed at the Research Technology Support Facility (RTSF), Michigan State University, East Lansing, MI, USA. The RNA was checked for integrity before library preparation and sequencing using a Bioanalyzer (Agilent Technologies). Briefly, multiplex sequencing libraries were prepared using the Illumina TruSeq Stranded mRNA Library Prep Kit LT. After QC, all six libraries (three treatment and three controls) were combined into a single pool. This pool was loaded on one lane of an Illumina HiSeq 2500 Rapid Run flow cell (v1). Sequencing was performed in a PE100 format with Rapid SBS reagents. Base calling was done by Illumina Real Time Analysis (RTA) v1.17.21.3 and output of RTA was de-multiplexed and converted to Fastq format with Illumina Bcl2fastq v1.8.4.

### RNA Sequencing Data Analysis

Raw reads in FASTQ format obtained from the Illumina platform were assessed for quality via RSeQC (v2.3.7). Sequence contamination was screened by Multi Genome Aligner (MGA v1.3^1^), which indicated more than 90% of all reads mapped to *O. sativa* MSU Genome v7^2^ with the majority of other reads not mapping to any species including 1000s of viruses, bacteria, plasmids as well as human and *Arabidopsis*. Raw reads were then aligned to the above reference genome using Rsubread (v1.16.1) (Liao et al., 2013), quantified via the function *feature Counts*, and voom transformed prior to differential expression with limma v3.20.9) (Law et al., 2014; Ritchie et al., 2015). Junctions were assessed via alignment with the function subjunc.

### Reverse Transcription Polymerase Chain Reaction

To validate the results of RNA-sequencing, we used reverse-transcriptase PCR (RT-PCR) for a selected subset of genes to determine their expression pattern under 1-, 7-, and 14-days post-treatment (dpt) with 50 μM 2,4-D. RNA samples were processed with the Ambion^®^ DNA-free DNase Treatment and Removal kit (Cat #AM1906, Foster City, CA, USA). First strand cDNA was synthesized from 300 ng of RNA using a Thermo Scientific RevertAid RT Kit (Cat #K1691, Wilmington, DE, USA) with Oligo(dT)_18_ primers per manufacturer’s instructions. Target genes were selected based on their fold change values from our RNA-Seq data as well as biological function. Using Integrated DNA Technologies (IDT) (Coralville, IA, USA) primer design software and BLAST (NCBI), intron-spanning gene-specific primer sets were designed for these selected genes and RT-PCR conditions were optimized for gene primers. *Cyclophilin* and *Actin* were used as internal reference genes in these experiments.

## Author Contributions

Conceived and designed the experiments: AM. Performed the experiments: RH, JT, HM, and AS. Analyzed the data: RH, JT, HM, KD, MW, and AM. Contributed reagents/materials/analysis tools: AM. Wrote the paper: AM, RH, JT, and MW.

## Conflict of Interest Statement

The authors declare that the research was conducted in the absence of any commercial or financial relationships that could be construed as a potential conflict of interest.

## Footnotes

1 http://bibiserv.techfak.uni-bielefeld.de/mga/

2 http://rice.plantbiology.msu.edu/

## Supplementary Material

FIGURE S1 **Lateral root numbers in rice and *M. truncatula* roots during 2,4-D treatment. (A,B)** Show that lateral root numbers are decreased in rice roots upon 2,4-D treatment when compared to controls at 7 and 14 dpt respectively. Data represents the average of two experimental replications (*n* = 20–25) ± SE. Asterisk (^∗^) denotes significant difference between the two conditions by *t*-test (*P* < 0.001). **(C,D)** Show that lateral root numbers are decreased in *M. truncatula* roots upon 2,4-D treatment when compared to controls at 7 and 14 dpt respectively. Data represents the average of two experimental replications (*n* = 40–45) ± SE. Asterisk (^∗^) denotes significant difference between the two conditions by *t*-test (*P* < 0.005).

FIGURE S2 **Nodule-like structure formation in *Medicago cre1* mutants using 2,4-D. (A)** NLS can be induced in *cre1-1* and *cre1-2* roots upon 2,4-D treatment at 7 dpt. However, the frequency of NLS formation in these mutants was significantly (one-way ANOVA, *F*_2,90_ = 3.4511, α = 0.05, *P* < 0.001) lower than in wild type plants. Data represents average of two experimental replications ± SE. **(B)** Average number of NLS formed per plant in wild type *M. truncatula* is higher than in *cre1-1* and *cre1-2* mutants at 7 dpt. Data represents average of two experimental replications ± SE. Asterisk (^∗^) denotes significant difference between the wild type and the mutants by *t*-test (*P* < 0.001).

FIGURE S3 **Gene ontology (GO) analysis of the differentially expressed TFs.** Differentially expressed transcription factors were subjected to singular enrichment analysis (SEA) in agriGO using default parameters. Bar chart of significantly enriched biological processes. Y-axis indicates the -log10 of the FDR adjusted *P*-value. Dotted red line indicates a FDR adjusted *P*-value = 0.05.

FIGURE S4 **Gene ontology analysis of the differentially expressed protein kinases.** Differentially expressed protein kinases were subjected to singular enrichment analysis (SEA) in agriGO using default parameters. Bar chart of significantly enriched biological processes. Y-axis indicates the -log10 of the FDR adjusted *P*-value. Dotted red line indicates a FDR adjusted *P*-value = 0.05.
