# Supplementary figures and images for: A Developmental and Molecular View of Formation of Auxin-Induced Nodule-Like Structures in Land Plants
Source: Front Plant Sci. 2016 Nov 11;7:1692. doi: 10.3389/fpls.2016.01692 (PMC5104908; doi:10.3389/fpls.2016.01692)

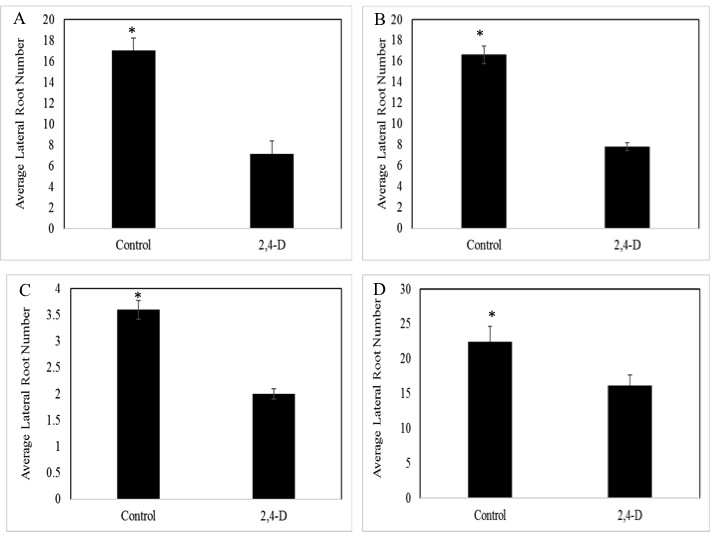

Supplement: FIGURE S1 — Lateral root numbers in rice and M. truncatula roots during 2,4-D treatment. (A,B) Show that lateral root numbers are decreased in rice roots upon 2,4-D treatment when compared to controls at 7 and 14 dpt respectively. Data represents the average of two experimental replications (n = 20–25) ± SE. Asterisk (∗) denotes significant difference between the two conditions by t-test (P < 0.001). (C,D) Show that lateral root numbers are decreased in M. truncatula roots upon 2,4-D treatment when compared to controls at 7 and 14 dpt respectively. Data represents the average of two experimental replications (n = 40–45) ± SE. Asterisk (∗) denotes significant difference between the two conditions by t-test (P < 0.005). [file Image_1.JPEG]

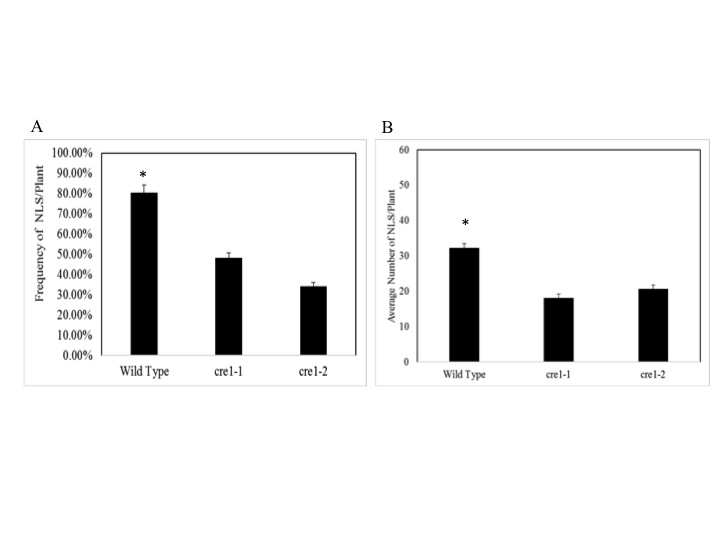

Supplement: FIGURE S2 — Nodule-like structure formation in Medicago cre1 mutants using 2,4-D. (A) NLS can be induced in cre1-1 and cre1-2 roots upon 2,4-D treatment at 7 dpt. However, the frequency of NLS formation in these mutants was significantly (one-way ANOVA, F2,90 = 3.4511, α = 0.05, P < 0.001) lower than in wild type plants. Data represents average of two experimental replications ± SE. (B) Average number of NLS formed per plant in wild type M. truncatula is higher than in cre1-1 and cre1-2 mutants at 7 dpt. Data represents average of two experimental replications ± SE. Asterisk (∗) denotes significant difference between the wild type and the mutants by t-test (P < 0.001). [file Image_2.JPEG]

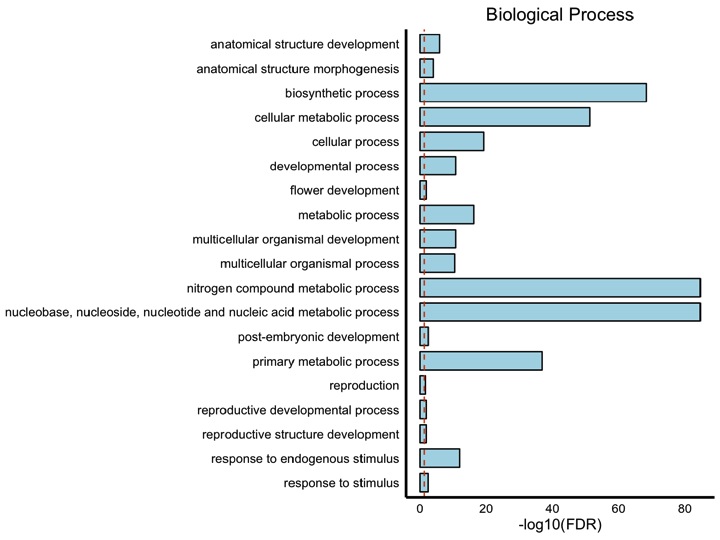

Supplement: FIGURE S3 — Gene ontology (GO) analysis of the differentially expressed TFs. Differentially expressed transcription factors were subjected to singular enrichment analysis (SEA) in agriGO using default parameters. Bar chart of significantly enriched biological processes. Y-axis indicates the -log10 of the FDR adjusted P-value. Dotted red line indicates a FDR adjusted P-value = 0.05. [file Image_3.JPEG]

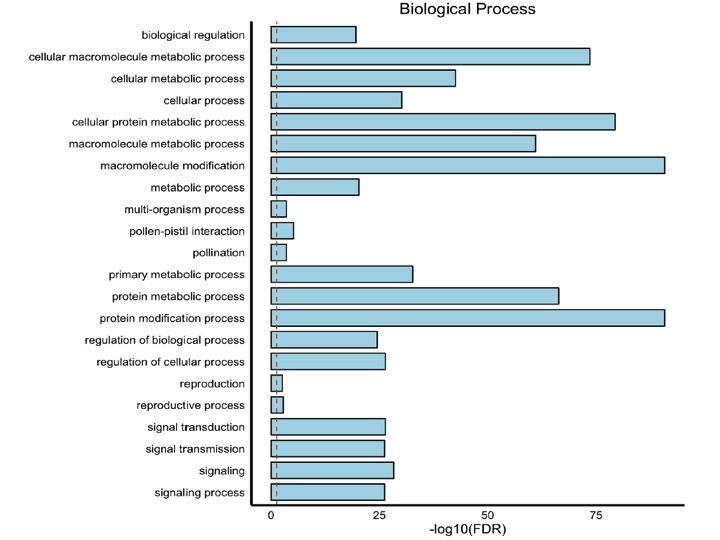

Supplement: FIGURE S4 — Gene ontology analysis of the differentially expressed protein kinases. Differentially expressed protein kinases were subjected to singular enrichment analysis (SEA) in agriGO using default parameters. Bar chart of significantly enriched biological processes. Y-axis indicates the -log10 of the FDR adjusted P-value. Dotted red line indicates a FDR adjusted P-value = 0.05. [file Image_4.JPEG]
